# Event-Based Gesture Recognition With Dynamic Background Suppression Using Smartphone Computational Capabilities

**DOI:** 10.3389/fnins.2020.00275

**Published:** 2020-04-09

**Authors:** Jean-Matthieu Maro, Sio-Hoi Ieng, Ryad Benosman

**Affiliations:** ^1^Sorbonne Université, INSERM, CNRS, Institut de la Vision, Paris, France; ^2^CHNO des Quinze-Vingts, INSERM-DGOS CIC 1423, Paris, France; ^3^Departments of Ophthalmology/ECE/BioE, University of Pittsburgh, Pittsburgh, PA, United States; ^4^Department of Computer Science, Robotics Institute, Carnegie Mellon University, Pittsburgh, PA, United States

**Keywords:** gesture recognition, event-based, neuromorphic, background suppression, smartphone, dynamic vision sensor (DVS), dynamic gesture recognition, mobile device

## Abstract

In this paper, we introduce a framework for dynamic gesture recognition with background suppression operating on the output of a moving event-based camera. The system is developed to operate in real-time using only the computational capabilities of a mobile phone. It introduces a new development around the concept of time-surfaces. It also presents a novel event-based methodology to dynamically remove backgrounds that uses the high temporal resolution properties of event-based cameras. To our knowledge, this is the first Android event-based framework for vision-based recognition of *dynamic* gestures running on a smartphone without off-board processing. We assess the performances by considering several scenarios in both indoors and outdoors, for static and dynamic conditions, in uncontrolled lighting conditions. We also introduce a new event-based dataset for gesture recognition with static and dynamic backgrounds (made publicly available). The set of gestures has been selected following a clinical trial to allow human-machine interaction for the visually impaired and older adults. We finally report comparisons with prior work that addressed event-based gesture recognition reporting comparable results, without the use of advanced classification techniques nor power greedy hardware.

## 1. Introduction

**Figure 1:**
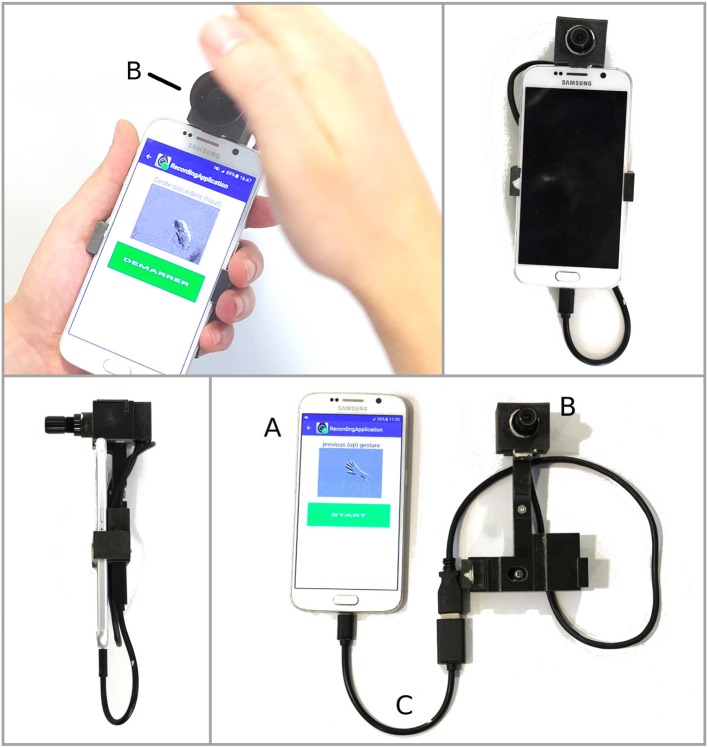
A neuromorphic camera (an ATIS) **(B)** is plugged into a smart-phone **(A)** using an USB link **(C)**, allowing mid-air gesture navigation on the smart-phone.

This article focuses on the problem of gesture recognition and dynamic background suppression using the output of a neuromorphic asynchronous event-based camera (Figure 1) connected to a mobile phone (Maro et al., 2019). The system does not rely on off-board resources. Event-based cameras (Lichtsteiner et al., 2008; Delbruck et al., 2010; Posch et al., 2011) offer a novel path to computer vision by allowing to operate at high temporal precision at equivalent frame rates at the order of several kilohertz. Contrary to standard frame-based cameras, which have a pre-defined acquisition rate, individual pixels of neuromorphic cameras are independent and react to relative changes of illuminance in their own field-of-view. Event-based cameras are scene dependent and therefore burn very little power depending on the amount of recorded data (1–10 mW). They hold the promise of low computational costs while operating at high temporal scales. However, there has been no development of a proof of concept using these properties in the context of edge computation. In this paper, we introduce a working prototype of a mobile phone event-based application. We chose the popular task of vision-based gesture recognition and dynamic background suppression. These are good targets to make use of the dynamic properties of event-based sensors. We chose to use a scalable machine learning architecture relying on the concept of time-surfaces introduced in Lagorce et al. (2016) and extended it to operate on the limited available computational resources. The system has been designed to operate on each incoming event rather than creating frames from the output of the sensor to then send them to a GPU.

Compared to previous event-based approaches that tackled the problem of gesture recognition, we emphasize the importance of using the information carried out by the timing of past events to obtain a robust low-level feature representation to avoid binning events into frames. We also address the difficult problem of dynamic background suppression by introducing a novel low power event-based technique operating in the temporal domain. This technique goes beyond existing background suppression methodologies. It uses the properties of data-driven acquisition and its high temporal resolution to segment a scene by setting a relation between depth and relative activity, thus allowing the foreground and background to be differentiated.

We also introduce a new dataset of gestures (*NavGesture*) recorded using an event-based camera and available for public download. The neuromorphic field still lacks datasets that take full advantage of the precise timing of event-based cameras. Available datasets such as N-MNIST and N-Caltech101 (Orchard et al., 2015a) are recording scenes where dynamics are artificially introduced. Even true neuromorphic datasets such as Poker-DVS (Serrano-Gotarredona and Linares-Barranco, 2015) or N-Cars (Sironi et al., 2018) contain limited *intrinsic* dynamic properties that could be used for classification. We intend to observe objects that can be classified using only their dynamic properties (or motion) and not from their spatial distribution. As an example, if one considers the N-Cars (Sironi et al., 2018) database, most objects appear as "flashes" that provide a snapshot of the object to be recognized. The DvsGesture dataset (Amir et al., 2017) fulfills the requirement of having dynamic properties, however the camera is set static with the same centring for all samples with no activity in the background. The American Sign Language dataset, ASL-DVS (Bi et al., 2019) offers various centring and scales but aims to recognizing hand postures and also lacks dynamic properties. The proposed dataset (*NavGesture*) is a new step toward bridging the gap between laboratory-recorded datasets and everyday real situations. It features a challenging set of dynamic gestures to classify, with heterogeneous centring and scaling using a moving camera both in indoor and outdoor environments.

### 1.1. Gesture Recognition on Mobile Devices

Gesture recognition on mobile devices is a quickly expanding field of research that uses a variety of sensors and methods (Pisharady and Saerbeck, 2015; Asadi-Aghbolaghi et al., 2017; Aditya et al., 2018). While resource-constrained devices such as smartphones disallow the use of certain technologies requiring high energy consumption such as vision-based depth (RGB-D) sensors, current mobile phones have a wide variety of built-in sensors. Several techniques use: phone speakers (Wang Z. et al., 2019), inertial sensors (Deselaers et al., 2015; Gupta et al., 2016; Li et al., 2018) or proximity sensors (Kim and Kang, 2010; Cheng et al., 2011). It is worth noticing that (Won et al., 2015) propose to use a neuromorphic camera as a proximity sensor instead of the conventional infra-red sensitive photo-diode. Other techniques use external components such as: e-gloves (Kau et al., 2015), radio-frequency chips (Kellogg et al., 2014) and even in some cases an external IMU for teeth gesture recognition (Gálvez et al., 2019).

Smartphones also use standard RGB cameras, allowing vision-based recognition. As pointed in Chakraborty et al. (2018), dynamic gestures must be captured at high frame rates in order to avoid motion blur and in some cases even missing a gesture. However, processing high frame rates video data in real time on a smartphone is computationally challenging if not impossible. This might explain why most if not all of the vision-based gesture recognition methods running on smartphones without off-board processing are only applied to static gestures (hand poses) (Ghanem et al., 2017; Lahiani et al., 2017). The only vision-based dynamic gesture recognition method for smartphone we found is proposed by Rao and Kishore (2016). However, no proof of concept operating on a mobile phone has been developed as the system has only been simulated on a resource-capped standard computer. Furthermore vision-based methods require to segment the hand from the background. This is often solved either by background pre-sampling (Dadiz et al., 2017) or by using skin color calibration (Jin et al., 2016; Lahiani et al., 2016). We will shortly show that this can be performed differently if one considers the high temporal resolution of event-based cameras.

### 1.2. Gesture Recognition Using Event-Based Cameras

Neuromorphic cameras coupled with event-based processing open new perspectives for resource management as both computation and memory can be allocated only to active parts of a visual scene. In the past few years a large number of work tackled computer vision problems using event-based cameras while keeping in mind the necessity of avoiding at all costs the temptation to generate frames from the sensor's output, to cite a few: optical flow estimation (Benosman et al., 2014), high-speed tracking (Serrano-Gotarredona et al., 2009; Ni et al., 2012; Valeiras et al., 2015), object classification (Sheik et al., 2013; Lagorce et al., 2015; Orchard et al., 2015b), 3D reconstruction (Ieng et al., 2018), or pose estimation (Reverter Valeiras et al., 2016).

Generating images from the output of event-based cameras to take advantage of decades of standard computer vision research is becoming a popular stream of research (Kogler et al., 2009; Mueggler et al., 2015; Pradhan et al., 2019; Rebecq et al., 2019). This has lead to the development of pipelines that convert conventional frame-based datasets into events either using hardware (Orchard et al., 2015a; Hu et al., 2016; Wang Y. et al., 2019) or software (Chadha et al., 2019). These data are then often converted back into frames in order to use frame-based techniques such as CNN. There is currently a need to carry out research on event-by-event processing to take full advantage of all the properties of neuromorphic vision sensors (Cadena et al., 2016; Chen et al., 2019). These sensors cannot only be used to generate high frame rates or high dynamic range images as one loses all advantages of the sparseness and low computation power associated to event-based acquisition.

To our knowledge, the first gesture recognition system using a Dynamic Vision Sensors (DVS) is the Rock-Scissor-Paper game from Ahn et al. (2011), which detected the final static hand pose using event activity. Samsung has developed several gesture recognition systems. In early experiments, they proposed to use Leaky Integrate-and-Fire (LIF) neurons to correlate space-time events in order to extract the trajectory of gestures, using a stereo-pair of DVS in Lee J. et al. (2012); Lee et al. (2014). This method is also adapted to track a finger tip using a single DVS (Lee J. H. et al., 2012), and event activity rate is also used to discriminate finger tip movements from hand swipes. Samsung also proposed to use the Adaptive Resonance Theory (ART) for continuous gesture recognition, first with HMM (Park et al., 2012), then with CNN (Park et al., 2015). In parallel to the trajectory extraction approaches, global motion-based features were proposed. Kohn et al. (2012) proposed a motion-based analysis of body movements using the relative event activity accumulated into 40 ms frames, while Lee K. et al. (2012) used pseudo optical-flow. To cope with varying speeds, Clady et al. (2016) proposed a motion-based feature that decays depending on the speed of the optical flow. Two end-to-end neuromorphic systems for gesture recognition have been proposed in recent years. The first one used the SpiNNaker neuromorphic board (Liu and Furber, 2015) and the second was implemented by IBM Research on the TrueNorth neuromorphic chip (Amir et al., 2017). However, both systems bin events into frames at some point in order to use a CNN for classification. Along with their implementation IBM has also released the DvsGesture dataset, which has become widely used in the neuromorphic community. It has been used in multiple papers: spatio-temporal filters that feed a CNN (Ghosh et al., 2019), SNN (Kaiser et al., 2018; Shrestha and Orchard, 2018), and a PointNet adaptation (Wang Q. et al., 2019).

Sign Language recognition has also been investigated but with a focus on static hand postures using events-to-frame techniques (Rivera-Acosta et al., 2017) or a graph-based CNN (Bi et al., 2019). Chen et al. (2019) proposed a new representation called *Fixed Length Gist Representation* (FLGR), mapping events to a higher dimensional feature. All presented methods used data from a static neuromorphic camera, with no background clutter. Furthermore, centring and scaling is in general the same except for (Bi et al., 2019). The only work to our knowledge with a focus on cluttered background and featuring one to several subjects *per se* quence, is the hand detection method proposed by Li et al. (2017). Unfortunately, they did not release their dataset. Also, it is worth mentioning that almost all presented works use at some point an events-to-frame conversion such as temporal or index binning, pixel spike rate or global memory surfaces. The only methods that process events in an event-based manner are scarce: (Lee J. H. et al., 2012; Lee K. et al., 2012), Clady et al. (Clady et al., 2016), SLAYER (Shrestha and Orchard, 2018) and FLGR (Chen et al., 2019).

In this work, we will consider more general scenarios offered by a moving camera that induces numerous new issues to solve such as: a higher number of emitted events, heterogeneous centering and scaling, unwanted shaking and important background clutter. Eliminating the background is an important step for event by event processing. Kyung et al. (2014) proposed a background suppression method for neuromorphic cameras, but converted events to frames. Our approach is purely event-based and drastically contrasts from any existing background removal algorithm as it uses only the timing of events and it does not rely on conventional approaches such as: code-books (Elgammal et al., 2000), probabilistic approaches (Stauffer and Grimson, 1999), sample-based methods (Barnich and Droogenbroeck, 2011), subspace-based techniques (Oliver et al., 2000), or even deep learning (Babaee et al., 2018).

## 2. Event-based Cameras and the Event-based Paradigm

**Figure 2:**
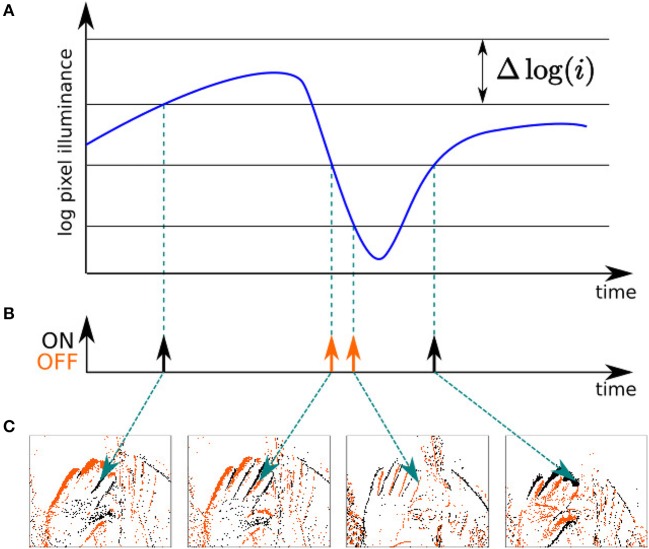
Principle of operation of the neuromorphic camera used in this work. **(A)** When the change in illuminance of a given pixel's field of view exceeds a certain threshold, **(B)** it emits a visual event, which is either "ON" or "OFF" depending on the sign of the change. **(C)** A given pixel responds asynchronously to the visual stimuli in its own field of view.

The Address Event Representation (AER) neuromorphic camera used in this work is the Asynchronous Time-based Image Sensor (ATIS) (see Figure 1B) (Posch et al., 2011). Each pixel is fully autonomous, independent, and asynchronous, it is triggered by a change in contrast within its field of view. A pixel emits a visual event when the luminance change exceeds a certain threshold, typically around 15% in contrast. The nature of this change is encoded in the *polarity* *p* of the visual event, which can be either ON (*p* = 1) or OFF (*p* = 0), depending on the sign of the luminance change (see Figure 2). We must emphasize that *p* does not carry meaningful information *per se*: indeed, a given object can induce both polarities depending on if the background is lighter or darker than the observed object. Hence, the polarity is context-dependant and can not be taken into account except in the case of a controlled environment and stimulus. The ATIS has a high temporal precision, in the order of hundreds of microseconds, which allows the capture of highly dynamical scenes while avoiding motion blur (Mueggler et al., 2014). The *k*-th visual event *e*_*k*_ of the output stream of the camera can be mathematically written as the following triplet:

(1) $$\begin{array}{r} e_{k}=\left(x_{k},t_{k},p_{k}\right) \end{array}$$

where *x*_*k*_ is the spatial location of the visual event on the focal plane, *t*_*k*_ its time-stamp, and *p*_*k*_ its polarity.

## 3. Methods

### 3.1. Dynamic Background Suppression

**Figure 3:**
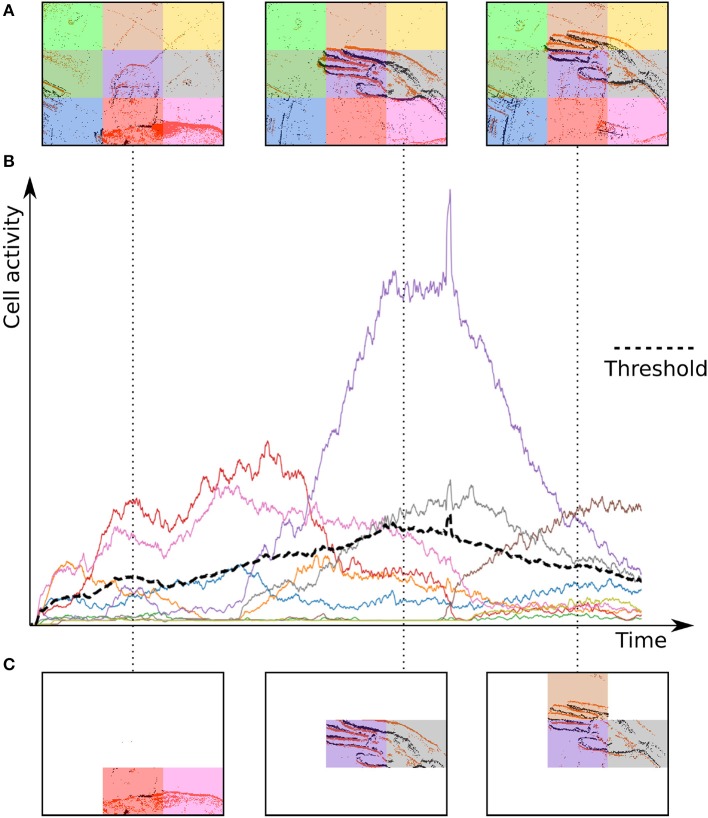
Operating principle of the Dynamic Background Suppression (DBS). **(A)** A gesture is performed in front of the camera, which pixel array is divided into cells. **(B)** Each cell has its own activity counter that decays over time. **(C)** Only cells with their activity greater than the mean activity (black dashes) of all cells can spike.

The Dynamic Background Suppression (DBS) uses the simple idea that the closer an object is to the camera, the more events it will generate as its apparent motion will be more important than a farther object. From this property it is possible to link the relative local activity within the focal plane to depth. A low event relative activity can be associated to the background and hence dismissed, whereas relative high activity regions could correspond to the foreground. Although the technique could be applied to each pixel, we will estimate the relative activity considering portions of the focal plane that will be divided into a grid of cells, as shown in Figure 3.

Let each cell *c* be composed of a set of pixels where activity is expressed by *A*_*c*_. For each incoming event *e*_*k*_ = (*x*_*k*_, *t*_*k*_, *p*_*k*_) emitted by a pixel belonging to a cell *c*, we can apply the following update of its activity *A*_*c*_ as:

(2) $$\begin{array}{r} A_{c}\leftarrow A_{c}\cdot \exp\left(-\frac{t_{k}-t_{c}}{\tau_{b}}\right)+1 \end{array}$$

where *t*_*k*_ is the time-stamp of the current event *e*_*k*_, *t*_*c*_ the last time *c* has been updated, and τ_*b*_ is a decaying time-constant.

**Figure 4:**
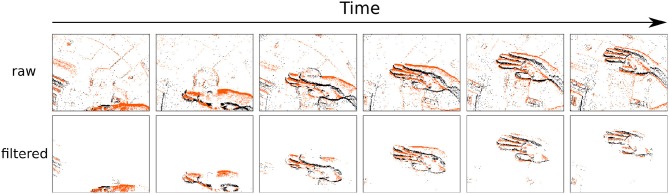
Denoising example of a gesture clip from the NavGesture-walk data-set. The presented gesture is a "swipe down". Top row is the raw stream of visual events, and the bottom row is the denoised stream, at the output of the 3rd stage of the cascade presented in this paper. Each snapshot from the top row is made of 10,000 events, and bottom row contains only the kept events of those 10,000. "ON" events are orange, "OFF" events are black. The filtering lead to the removal of 83.8% of all events. Even after removing this many events each gesture is still easily recognizable by the human eye.

We can then compute the average activity $\bar{A}$ of a all cells. An incoming event *e*_*k*_ = (*x*_*k*_, *t*_*k*_, *p*_*k*_) belonging to *c* is sent to the machine learning module only if:

(3) $$\begin{array}{r} A_{c}\geq \max\left(\alpha \bar{A},A_{T}\right) \end{array}$$

where α is a scalar to set the aggressiveness of the filter, and *A*_*T*_ is a threshold for minimum foreground activity. The activity of a cell and the threshold $\bar{A}$ are computed for each incoming event, which enables or disables a given cell at the temporal resolution of incoming events. Cells with a low activity are considered as background and are prevented from emitting events. In principle each time a cell is updated the general mean activity has to be updated. Events are timed at the μ*s* and are orders of magnitude faster than any conventional urban real scene dynamics. The mean activity can then be updated at much lower temporal scales set experimentally according to the computation power available and perhaps the situation (one can infer acceleration from the built-in IMU). The proof of principle of the technique is shown in Figure 4 and an example of a denoised clip is provided in Video S1.

### 3.2. Time-Surfaces as Spatio-Temporal Descriptors

A time-surface (Lagorce et al., 2016) is a descriptor of the spatio-temporal neighborhood around an incoming event *e*_*k*_. We define the time-context *T*_*k*_(*u, p*) of the event *e*_*k*_ as a map of time differences between the time-stamp of the current event and the time-stamps of the most recent events in its spatial neighborhood. This (2*R* + 1) × (2*R* + 1) map is centered on *e*_*k*_, of spatial coordinates *x*_*k*_. The time-context can be expressed as:

(4) $$\begin{array}{r} T_{k}\left(u,p\right)=\left\{t_{k}-t|t=\underset{j\leq k}{\max}\left\{t_{j}|x_{j}=\left(x_{k}+u\right),p_{j}=p\right\}\right\} \end{array}$$

where $u={\left[u_{x},u_{y}\right]}^{T}$ is such that *u*_*x*_ ∈ ⟦−*R, R⟧* and *u*_*y*_ ∈ ⟦−*R, R⟧*.

Finally, we obtain the time-surface *S*_*k*_(*u, p*) associated with the event *e*_*k*_, by applying a linear decay kernel of time-constant τ to the time-context *T*_*k*_:

(5) $$\begin{array}{r} S_{k}\left(u,p\right)=\{\begin{array}{ll} 1-\frac{T_{k}\left(u,p\right)}{\tau }, & \text{if}T_{k}\left(u,p\right)<\tau \\ 0, & \text{otherwise} \end{array} \end{array}$$

**Figure 5:**
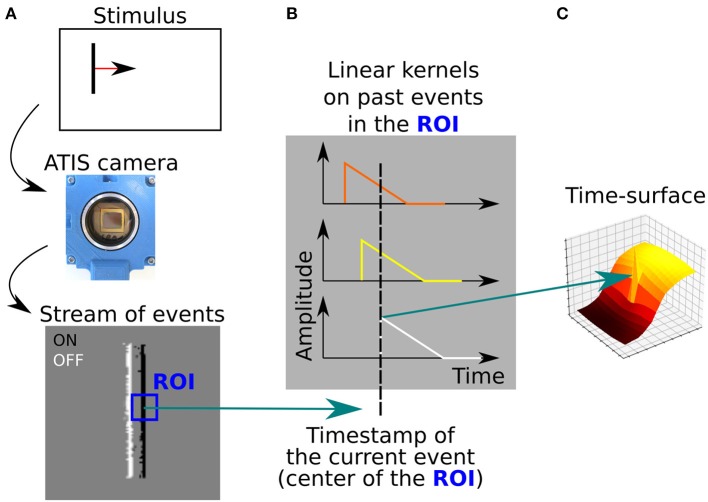
**(A)** A moving vertical bar is presented to the event-based camera, which output a stream of visual events. The edges of the bar are ON (white) and OFF (black) events. A ROI is defined around the current event (blue square). **(B)** The time-stamps of visual events contained the ROI are decayed using a linear kernel. **(C)** The resulting extracted time-surface, that encodes both the contour orientation and the dynamic of the motion.

*S*_*k*_ is a low-level representation of the local spatio-temporal neighborhood of the event *e*_*k*_. Figure 5 illustrates how time-surfaces are computed from the stream of events.

**Discarding time-surfaces**. A time-surface can be computed for each new incoming event, but would generate overlapping time-surfaces and introduce redundancy. As the event-based camera performs native contour extraction, we must ensure that a sufficient number of events to form a full contour are taken into account. Therefore, time-surfaces must be discarded if they contain too little information, using the following heuristic:

(6) $$\begin{array}{r} card\left(\left\{\left(u,p\right),T_{k}\left(u,p\right)<\tau \right\}\right)\geq 2R \end{array}$$

### 3.3. Event-Based Hierarchical Pattern Matching

Following the principle of using deep multiple temporal and spatial scales introduced in HOTS (Lagorce et al., 2016), incoming visual events are fed to a network composed of several layers. As events flow into the network, only their polarities are updated on successive "feature planes." Polarities in the network correspond to learned patterns or elementary features at that temporal and spatial scale. However, as time-surfaces can be discarded, the network output stream contains less events than the input stream, which is an important property that builds on the native low output of the event-based camera to lower the computational cost.

#### 3.3.1. Creating a Layer and Learning Prototypes

An iterative online clustering method is used to learn the base patterns (hereinafter called prototypes), as it allows to process events as they are received, in an event-based manner. A layer is composed of a set of *N* prototypes, which all share the same radius *R* (which corresponds to the neuron's receptive field), and the same time-constant τ. The triplet (*N, R*, τ) defines a layer. First, a set of *N* time-surface prototypes *C*_*i*_, with *i* ∈ ⟦0, *N* − 1⟧, is created. The *C*_*i*_ are initialized by using random time-surfaces obtained from the stream of events. For each incoming event *e*_*k*_ we compute its associated time-surface *S*_*k*_ of radius *R* and time-constant τ. Using the L2 Euclidean distance, we compute the closest matching prototype *C*_*i*_ in the layer, which we update with *S*_*k*_ using the following rule, improved from Lagorce et al. (2016):

(7) $$\begin{array}{r} C_{i}\leftarrow C_{i}+\alpha_{i}\frac{S_{k}\cdot C_{i}}{\left||S_{k}||||C_{i}|\right|}\left(S_{k}-C_{i}\right) \end{array}$$

with α_*i*_ the current learning rate of *C*_*i*_ defined as:

$$\alpha_{i}=\frac{1}{1+A_{i}}$$

where *A*_*i*_ is the number of time-surfaces which have already been assigned to *C*_*i*_. If a prototype *C*_*i*_ is poorly triggered, it is re-initialized and forced to learn a new pattern. This prevents badly initialized prototypes to stay unused, and helps them converge to meaningful representations.

#### 3.3.2. Building the Hierarchy

**Figure 6:**
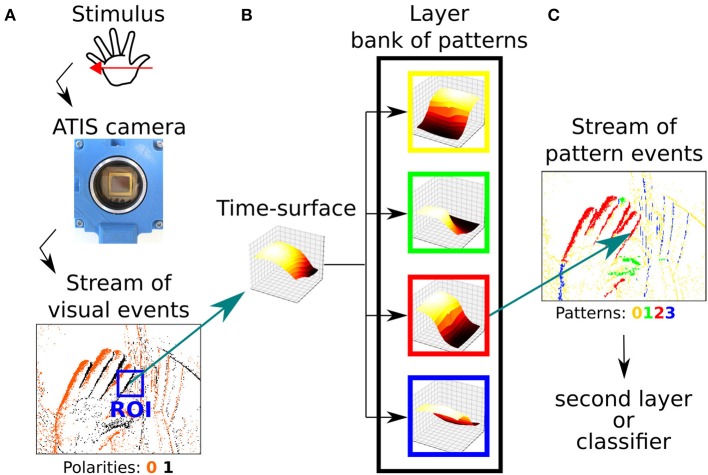
**(A)** A stimulus is presented in front of a neuromorphic camera, which encodes it as a stream of event. **(B)** A time-surface can be extracted from this stream. **(C)** This time-surface is matched against known pattern, which are also time-surfaces, and that can be used as features for classification.

One can then stack layers in a hierarchical manner, in order to form a network (see Figure 6). First, the visual stimulus is presented to the event-based camera (Figure 6A), which outputs a stream of visual events. A given event *e*_*m*_ of the stream must go through all the layers before the next event *e*_*m* + 1_ is processed. At each layer (*N, R*, τ), if the time-context *T*_*m*_ of the event *e*_*m*_ satisfies Equation (6), the corresponding time-surface *S*_*m*_ is computed (see Figure 6B). Then, the best matching prototype *C*_*c*_ is updated using Equation (7) (see Figure 6B). At this point, the polarity *p*_*m*_ of *e*_*m*_ is modified so that *p*_*m*_ = *c*, *c* being the ID of the best matching prototype. Event *e*_*m*_ is then sent to the next layer to be processed in a similar manner. We must emphasize that the first layer, which receives *visual events* from the camera does not take the polarity (that corresponds to the increase or decrease in contrast) into account for the reason exposed in section 2. All *visual events* have their polarity *p* set to zero. In the subsequent layers, however, the polarity now encodes a pattern, and we refer to them as *pattern events* instead of *visual events* for which the polarity corresponds to a luminance change. Pattern events are then fed to the next layer, and processed in a similar manner. As we go higher in the hierarchy of layers, subsequent layers combine patterns from previous layers, thus their prototypes (and so the corresponding polarities) encode more and more sophisticated patterns. As an illustration, the first layer can only encode the shape and the direction of the motion. The second layer however, because it is working with the first layer output can encode changes of direction in the motion. Once the full hierarchy has been trained, meaning that its time-surface prototypes have converged, the learning is disabled: prototypes are no longer updated using Equation (7).

The network can now serve as a feature extractor: the polarities of events output by the network will be used as features for classification. Because this algorithm is truly event-based and data-driven the computation time directly depends on the number of events transmitted by the camera.

## 4. A new neuromorphic dataset: NavGesture

As mentioned in the previous section, existing gesture and action recognition datasets are recorded using a non-moving camera set in front of a static background (Amir et al., 2017; Bi et al., 2019; Chen et al., 2019; Ghosh et al., 2019; Wang Y. et al., 2019). In some other popular neuromorphic datasets such as N-MNIST and N-Caltech101 (Orchard et al., 2015a), the event-based camera is set up on a pan-tilt in front of a computer screen, hence the dynamics of recorded objects correspond to the pan-tilt movement. The same issue arises in N-Cars (Sironi et al., 2018) because of the very short duration of each clip. Furthermore cars are cropped, removing most of the background.

The proposed dataset offers a challenging gesture recognition task because of its dynamic and changing backgrounds. All gestures were recorded in selfie mode, with the users holding the camera with one hand and performing the gesture with their free hand. The fact that users where holding the phone leads to a wide variety of centring and gesture distance to the camera. The dataset features both right-handed and left-handed users. The users were either sitting or walking, indoors and outdoors, in uncontrolled lighting conditions. The neuromorphic camera used is an ATIS (Posch et al., 2011) with a lens VM-6.5-IR-CCD from Universe Optics. This choice was made in order to facilitate the "auto"-centring by the end-users, by allowing a larger field of view.

The NavGesture dataset has originally been designed to facilitate the use of a smartphone by the elderly and the visually impaired. The gesture dictionary has 6 gestures in order to be easily memorized. They have been selected to be the most compact set able to operate a mobile phone. Four of them are "sweeping" gestures: *Right, Left, Up, Down*. These are designed to navigate through the items in a menu. The *Home* gesture, a "hello"-waving hand, can be used to go back to the main menu, or to obtain help. Lastly, the *select* gesture, executed only using fingers, closing them as a claw in front of the device, and then reopening them, is used to select an item.

The NavGesture dataset is split into two subsets, depending on whether users were sitting or walking: NavGesture-sit and NavGesture-walk. The NavGesture-sit dataset features 28 subjects, 12 being visually impaired subjects, with a condition ranging from 1 to 4/5 on the WHO blindness scale and 16 being people from the laboratory. The gestures were recorded in real use condition, with the subject sitting and holding the phone in one hand while performing the gesture with their other hand. Some of the subjects were shown video-clips of the gestures to perform, while others had only an audio description of the gesture. This inferred some very noticeable differences in the way each subject performed the proposed gestures, in terms of hand shape, trajectory, motion and angle but also in terms of the camera pose. Each subject performed 10 repetitions of the 6 gestures. In a second stage, all the acquired clips were manually labeled and segmented. We removed problematic clips, such as wrongly executed gestures or gestures executed too close to the camera. The manually curated dataset contains a total of 1, 342 clips.

In the NavGesture-walk the users walked through an urban environment, both indoors in the laboratory, and outdoors in the nearby crowded streets in the center of Paris. Users recorded the gestures while walking, holding the phone with one hand and performing the gestures with the other. This uncontrolled setting leads to much more variation in pose, unwanted camera movements, dynamic backgrounds and lighting conditions. This dataset features 10 people from the laboratory that performed 5 times each of the 6 gestures. The dataset contains a total of 339 clips. An overview is presented in Table 1. An example of the "Swipe Up" gesture is shown in Figure 4. The NavGesture dataset is publicly available at https://www.neuromorphic-vision.com/public/downloads/navgesture/.

**Table 1:** Characteristics of the three Gesture Datasets used in this work.

**Dataset**	**#users**	**#classes**	**#clips**	**Camera**	**Background**	**Framing**
DvsGesture	29	10 + 1	1,342 + 122	Static	No	Upper body
NavGesture-sit	28	6	1,342	Handheld	Yes, moderate	Selfie, user sitting
NavGesture-walk	10	6	339	Handheld	Yes, important	Selfie, user walking

## 5. Experiments and Results

The first experiment on the Faces dataset focuses on extracting static properties. We show that a single layer is sufficient to provide good results. The following experiments required more layers. As the neuromorphic camera detects change in contrast, these can either be ON or OFF events depending on the contrast between the foreground and the background. Indeed, the same moving object could generate ON events in front of a dark background, and OFF events in front of a light background, as explained earlier. This is the reason why in all the following experiments we did not take the polarity of visual events into account, as the polarity is context-dependent. An example of this phenomena is a moving hand in front of a black and white stripped background. This is why we considered that only the illuminance *change* carries information for these classification tasks, and not the fact that the illuminance *increased* (ON event) or *decreased* (OFF event).

For all classification tasks, the output of end-layers (larger time scale) is integrated over time to generate a histogram of activity per feature as in Lagorce et al. (2016). This histogram is then used as a dynamic signature of the observed stimulus. This signature is fed to a classifier, in this case a nearest neighbor. More sophisticated classifiers could be used, but this demonstrates that extracted features are sufficient for classification.

### 5.1. Static Properties: Experiments on the Faces Dataset

This dataset contains clips of the faces of 7 subjects. Each subject was recorded 24 times, resulting in 168 clips. The subjects had to move their head in a square-shaped trajectory, by following a dot on a computer screen. The dynamic is therefore the same for all subjects, and does not carry any meaningful information for the classification task. Experiments were performed on a standard desktop computer. We performed 10-fold cross-validation with 5 examples in the train subset, and 19 in the test subset. We used a single-layer with *N* = 32 prototypes, receptive fields of radius *R* = 6 and τ = 5 ms, we obtained 96.6% recognition score on this dataset. By increasing the number of prototypes to *N* = 64, we achieved 98.5% in average recognition rate. We noticed that increasing τ higher than 5 ms was not beneficial and even decreased our classification accuracy. This is because time-surfaces encode both static properties such as shape and dynamic properties such as optical flow. A small τ will mainly encode static properties whereas a larger τ will also encode dynamic properties such as pseudo optical-flow. When we added a second layer, the recognition rate dropped. A single layer is therefore sufficient to encode static properties such as shape. The classification was made using a 1-nearest neighbor, and does not rely on advanced classification techniques.

In comparison, the HOTS model in Lagorce et al. (2016) performed at 79% using a three-layer architecture, with its end-layer having *N* = 32 of prototypes. It must be noted that this improvement in recognition rate also comes with a faster computation because of the reduction in the size of used time-surfaces, from size 4,624 in HOTS to size 169 in our work.

Classification scores depend on the number of prototypes: the more prototypes, the higher the recognition rate.

### 5.2. Dynamic Properties: Experiments on the NavGesture Datasets

In both NavGesture-sit and NavGesture-walk datasets, subjects hold the phone in their hand, which results in camera movements and unwanted jitters that generate background activity. In the case of the NavGesture-walk the visual background is even more present as subjects are walking while performing the gestures. The experiments were performed on a standard desktop computer, and we used *k*-fold cross-validation, with *k* the number of subjects.

In order to remove events generated by the background we used the Dynamic Background Suppression method introduced in section 3.1. The DBS uses the following parameters, set experimentally:

- τ_*b*_ = 300μs
- α = 2
- *A*_*T*_ = 5
- grid size : 3 × 3

Figure 4 illustrates the effect of the DBS. Table 2 reports the mean percentage of remaining events for each gesture after removing the background. The DBS allows to remove around 40% of events before the feature extraction. This has a direct impact on processing time as we compute event by event.

**Table 2:** Mean percentage of events left after each the Dynamic Background Suppression for each gesture class.

**Gesture**	**Mean number of event**	**Mean percentage left after the DBS**
Down	988,901	41%
Home	2,398,850	48%
Left	969,014	42%
Right	962,501	43%
Select	1,212,222	30%
Up	1,110,652	44%

In our experiments we used networks composed of 1 to 3 layers. We observed that two-layers networks perform better. Some gestures such as "Select" or "Home" have changes in direction, which can be encoded by networks with two or more layers. However, we suspect that three-layers networks encode features that are too complex for the stimulus, resulting in less discriminative features and a lower recognition rate.

Because events are decayed over time, the value of τ must correspond to the dynamic of the stimulus (Clady et al., 2016). If τ is too small, the extracted time-surface will encode only spatial information. If τ is too large, the trail of older events will blur the shape, encoding only direction of movement. In more extreme cases with τ going to larger and larger values, the resulting time-surface will carry less and less information, as all past events will have the same weight. Of course this has also a close relation with the radius of the time-surface as larger radii can encode longer trails of events.

This observation leads to the fact that τ should be set in regard to the radius *R* of the time-surface and the velocity *v* of the apparent motion in pixel per second:

(8) $$\begin{array}{r} \tau \approx \frac{R}{v} \end{array}$$

We observed that a first layer with a τ value in the order of 10 ms allowed to encode both shape and direction of motion (only direction, not changes in direction). The second and end-layer has a τ value of 100 ms, in order to encode changes in the direction of motion.

A direct difficulty comes from the almost fish-eye field of view of the camera: if the phone is not held vertically or if the gesture is a bit off-axis, it becomes very difficult at the edges of the field of view to determine if the motion is vertical or horizontal.

**Ablation study**. In order to assess the benefits of the DBS in obtaining better recognition rates, we compared the performance achieved with and without the DBS. Results show that DBS does improve recognition rates, increasing the score from 81.3 to 92.6% when using the NavGesture-walk dataset, as shown in Table 3.

**Table 3:** Summary of obtained results on the NavGesture dataset.

**ID**	**Dataset**	**Layer 1**			**Layer 2**			**DBS**	**Classifier**	**Results**
		**N**	**R**	**τ**	**N**	**R**	**τ**			
E1	NavGesture-sit	8	2	10 ms	8	2	100 ms	✓	k-NN	95.9%
E2	NavGesture-walk	8	2	10 ms	8	2	100 ms	✓	k-NN	92.6%
E3	NavGesture-walk	8	2	10 ms	8	2	100 ms		k-NN	81.3%
E4	NavGesture-walk	8	2	10 ms				✓	k-NN	88.7%

*The use of the Dynamic Background Suppression in E2 allows to drastically improve the recognition rate by over 10% compared to E3. Also, the addition of a second layer is beneficial, as shown by the improvement in E2 compared to E4*.

### 5.3. Experiments on the DvsGesture Dataset

**Figure 7:**
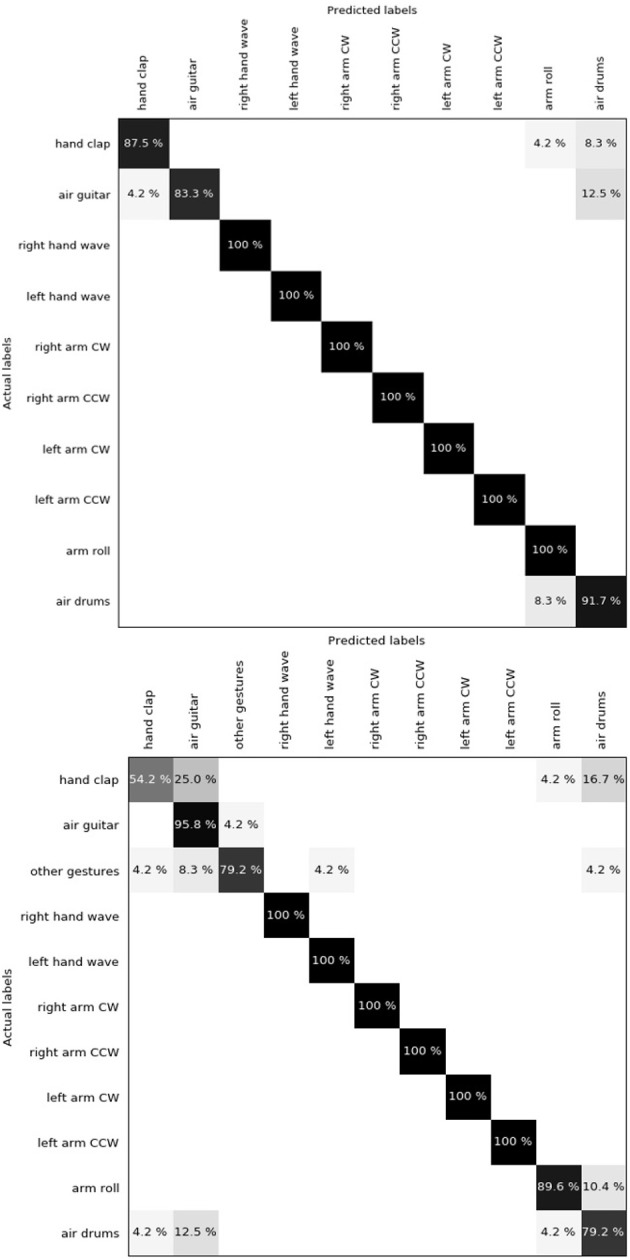
**(Top)** Confusion matrix for DvsGesture using 10 classes. Global accuracy is 96.59%. "Hand clap", "Arm roll", "Air guitar" and "Air drum" are the only gestures that get confused. The reason might be that they generate similar motion in the same spatial location. **(Bottom)** Introducing the rejection class "Other gestures" amplifies the mismatch between the four precedent gestures, leading to a global accuracy of 90.62%. However, it has almost no impact on other gestures (4.2% in the "Other gestures" row corresponds to only one clip).

Amir et al. (2017) released a 10-class (plus a rejection class with random gestures) dataset of hand and arm gestures, performed by 29 subjects under 3 different lighting conditions. The camera is mounted on a stand while the subjects stood still in front of it. This dataset has no background so the DBS was not used. Authors split the dataset into a training set of 23 subjects and a testing set of 6 subjects, preventing cross-validation for comparison purposes. We used the same 2-layer network architecture as the one used for NavGesture. The only difference is that we increased the number of prototypes in the last layer because the gestures are more complex. In order to take into account the spatial component of gestures, we split the pixel array into sub-regions, using a 3 × 3 grid. This is possible because the centring is very similar for all clips in the dataset. Hence, the final feature is a histogram of size 3 × 3 × 64 = 576. We achieved a classification accuracy of 96.59% for the 10-class subset and 90.62% for the 10 classes plus the rejection class. One can observe in the confusion matrix (Figure 7) that “Hand clap,” “Arm roll,” “Air guitar,” and “Air drum” are the only gestures that are mistaken. These gestures all share very similar hand movements at the same spatial location, located in front of the torso. “Arm roll” and “Air drum” are also very similar. Their difference lie in the fact that hands in “Arm roll” move along the same vertical line, and we suspect that the receptive field is too small to capture this information.

When adding the rejection class, the same gestures get confused. Indeed, only one clip of "Left hand wave" gets mistaken for "Air guitar", which is understandable as the left hand in these two classes performs the same movement at the same location. The global accuracy decreases mostly because of the "Hand clap" that gets misclassified more often and because of the "Other gestures" that also are harder to classify.

One can observe in Table 4 that for the 10-class classification task our system performs in the same range of accuracy using a k-NN as other very elaborate systems using state-of-the-art neural networks.

**Table 4:** Comparison in accuracy of state-of-the-art methods for the DvsGesture dataset.

	**Method**	**DvsGesture (10 classes)**	**DvsGesture (10 classes + 1)**
Amir et al. (2017)	CNN (avg 192 ms)	91.77% (96.49%)	91.77% (94.59%)
Shrestha and Orchard (2018)	SLAYER		93.64%
Kaiser et al. (2018)	DECOLLE		94.18%
Ghosh et al. (2019)	ST filter + CNN (avg 200 ms)		94.85% (95.94%)
Kaiser et al. (2019)	SNN eRBP		92.7%
Wang Q. et al. (2019)	PointNet++ (avg 118 ms)	96.34% (97.08%)	94.10% (95.32%)
This work	Time-surfaces + k-NN	96.59%	90.62%

*When noted (avg) an averaging scheme was proposed to improve the system accuracy. Our method, although using a simple k-NN classifier performs in the same range for the 10-class classification. However, the k-NN lacks the discriminative power to handle the rejection class on the contrary of more sophisticated classifiers*.

It must be noted that the same time constants gave best results for both NavGesture and DvsGesture, which shows that decay must be chosen in accordance with the stimulus, in both case gestures. Indeed, previous work such as HOTS (Lagorce et al., 2016) and (Sironi et al., 2018) used decay times that were three orders of magnitude higher than the duration of the stimulus. This resulted in time-surfaces that acted as binary frames instead of encoding the dynamics of the scene. Furthermore, such high decay values resulted in the incapacity of forgetting past events.

## 6. Implementation on a Smartphone

**Figure 8:**
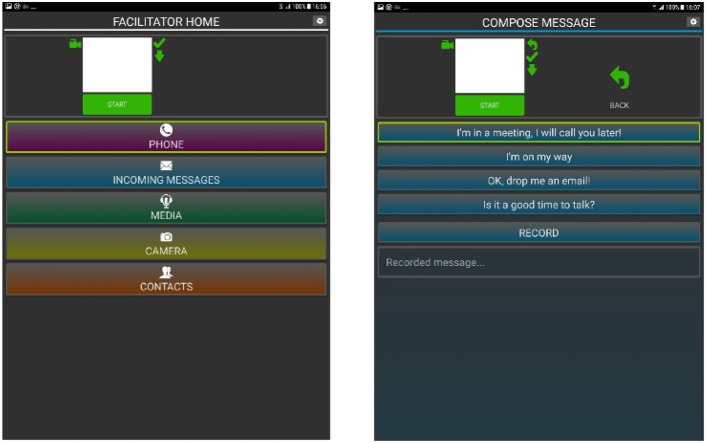
Interface of the Android application that was developed in order to operate the phone using the proposed gestures. Right is the main menu, left illustrates the pre-defined messages the user could send.

The proposed gesture recognition pipeline has been implemented on a mobile phone (Maro et al., 2019), a Samsung Galaxy S6 (model GM-920F), with a custom Android application allowing easy navigation through basic phone functions, such as making a call or sending a pre-defined text message (see Figure 8). The event-based camera was directly plugged into the micro-USB port of the mobile phone (see Figure 1). The gesture recognition module is implemented in native C++ using JNI to communicate with the Android application. The gesture recognition module consists of basic noise filtering (a refractory period followed by a spatio-temporal denoiser, known as the *background activity filter*, that removes pixel electrical noise), the Dynamic Background Suppression, a 1-layer Feature Extractor (*N* = 8, *R* = 2, τ = 10 ms,) and a k-NN classifier.

**Figure 9:**
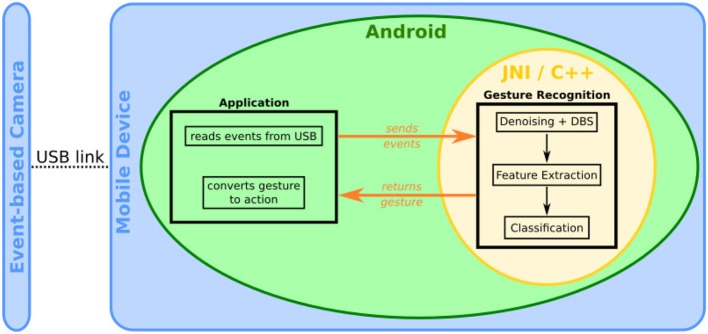
Overview of the Android smartphone system.

We used two strategies to segment gestures, the first one is an "auto-start" based on the global visual scene activity. This option works when users are seated but is inadequate for walking cases. The second strategy relied on pressing a button before a gesture to start the recording. The duration of the recording was tuned experimentally to 2 s which seems to be the experimental upper bound of the duration of a gesture. This 2-s batch of events at once to the gesture recognition module, that returns the gesture class to the Android application to be converted to an Android command. An overview of the system is presented in Figure 9.

To assess processing time, we ran five trials for each gesture in two different settings. The input event stream having a duration of 2 s, a real-time processing is reached when the processing time is below 2 s. In the first scenario, the phone was set on a table. In the second scenario the phone was handheld in selfie mode, with the user walking around. All results are compiled in Table 5. When looking at the first scenario, we can see that all gestures are under the 2 s barrier, except for the "Home" gesture (a "Hello-waving" gesture). This is because this gesture produces 3 times more events than all other gestures (see Table 2). The algorithm being truly event-based, the processing time directly depends on the number of events to process. Also during trials 3 and 4, the user waved his hand 5, 6 times, while in trials 1, 2, and 5 waved only 3, 4 times. The second scenario is the handheld selfie mode scenario, where the background generates a high number of events, hence necessitating longer processing time. However, all gestures except for the gesture "Home" that could be computed in real-time. This gesture should be replaced by another more event-based friendly gesture that would generate less events, or should be more constrained by forcing users to only wave 1 or 2 times.

**Table 5:** Processing time in milliseconds for five trials of each gesture on the mobile phone, depending on two conditions.

**Trial**	**Up**	**Home**	**Right**	**Left**	**Select**	**Down**
**Processing time in ms for 2,000 ms of input Setting: fixed position (no background)**						
1	132	2,343	54	127	40	54
2	57	2,798	60	56	57	45
3	74	3,047	44	275	61	42
4	254	3,833	32	42	29	54
5	48	2107	28	45	47	51
**Processing time in ms for 2,000 ms of input Setting: outdoor - moving**						
1	320	4,119	154	641	138	115
2	614	3,669	704	282	265	451
3	468	4,305	854	421	551	342
4	569	3,681	575	548	956	371
5	899	3,890	722	354	892	620

*“Fixed position” corresponds to a mobile phone set on a table, which means no background. “Outdoor, moving” corresponds to handheld selfie mode, while walking around. Each gesture corresponds to 2,000 ms of events, meaning that except for the “Home” gesture, all proposed gestures can be processed on real-time. The event-based camera is data-driven so a gesture like “Home' which corresponds to several “swipe” gestures will generate more events (see Table 2). Our algorithm being truly event-based it is also dependent on the number of events, and takes more processing time the more events it receives*.

This prototype was tested by untrained visually impaired end-users, in real use conditions. The subjects were asked to perform certain tasks to operate the phone. These preliminary tests lead to a global accuracy of 78%, which is below the 88.7% accuracy we obtained using the same single layer on the NavGesture-walk dataset. We suspect this is partly due to framing and off-axis handling of the phone.

## 7. Discussion and Conclusion

This paper introduced a proof of concept for an event-based Android application for gesture recognition using the computing power of a mobile phone. The main idea was to show that it is possible to make full use of the high temporal resolution of event-based cameras on a power-constrained device. The system used a camera designed to operate with Android using the USB link to stream events. This is by far a very inefficient way to input data to the mobile platform as USB is often too slow and implies time stamping events that adds more bits of information to the acquired events. It is expected that if this type of camera is one day introduced in a mobile device it will use better connectivity such as MIPI buses which are designed for low-power applications and eventually an associated processor. This will remove the need for time stamping and allow both direct routing to the processor and direct computation on the time of arrival of events with no delays. In this paper due to the limitations of the developed software we used 2-s packets of events to optimize communication within the phone. However, we showed that processing required in most cases less than 2 s per batch, which implies that real time performance can be reached if transmission delays are solved. We are confident that a way can be found within Android to transmit events from the camera to the processing stage with no latency. We have also shown that it is possible to handle the stream of events in an asynchronous manner. This allows the temporal machine learning algorithm to be efficient while using only a single core of the phone. The hierarchical temporal network has been optimized for the set of defined gestures showing that robust recognition levels can be reached without requiring the use of GPU or using the non-event-based concept of generating frames from an event-based sensor. Experimental results show that as expected the computation is scene dependent and therefore tightly linked to the amount of events generated by the observed object.

We have also shown that the temporal precision of event-based cameras can tackle different tasks, where it would have been too computationally expensive or even impossible to compute with frames in an elegant and low-power manner. As an example, the background suppression algorithm that for the first time considers outdoor, hand-held scenarios relies on the simple idea that the foreground being closer to the camera will on average generate more events than the background. The idea of using the relative mean activity for background suppression shows that high temporal precision is a valuable feature as it implies that velocity is linked to the amount of data produced, and can be estimated precisely. Moreover, the use of well designed temporal filters can reduce even more the already sparse steam of events, leading to faster event-by-event computation.

There is still so much to develop around the concept of using time as a computational feature for mobile applications. As an example the use of scene dynamics allows to derive techniques such as the one in Lenz et al. (2018) that uses the temporal signature of eye blinks to detect the presence of a face in a scene. This approach introduces an alternative to the current greedy stream of thought that believes everything has to be learned using large databases.

All data collected and used in the paper has been made available to the community. The introduction of this new database will set the groundwork for further work on dynamic background suppression.

## Data Availability Statement

The datasets generated for this study are available on request to the corresponding author.

## Ethics Statement

Ethical review and approval was not required for the study on human participants in accordance with the local legislation and institutional requirements. The patients/participants provided their written informed consent to participate in this study. Written informed consent for participation was not required for this study in accordance with the national legislation and the institutional requirements. Written informed consent was obtained from the individual(s) for the publication of any potentially identifiable images or data included in this article.

## Author Contributions

J-MM compiled the new gesture databases, designed the theory for background suppression, designed the experiments, performed analysis. J-MM and RB interpreted data for gesture recognition. J-MM wrote the article. J-MM, S-HI, and RB helped to edit the manuscript.

### Conflict of Interest

The authors declare that the research was conducted in the absence of any commercial or financial relationships that could be construed as a potential conflict of interest.

## Supplementary Material

Video S1 Dynamic Background Suppression at work.
